# Simeprevir with peginterferon α-2a/ribavirin for chronic hepatitis C virus genotype 1 infection in treatment-experienced patients: an open-label, rollover study

**DOI:** 10.1186/s12879-017-2444-3

**Published:** 2017-06-02

**Authors:** Edward J. Gane, Edwin DeJesus, Ewa Janczewska, Jacob George, Moises Diago, Mariliza Hendrique Da Silva, Henk Reesink, Igor Nikitin, Holger Hinrichsen, Stefan Bourgeois, Peter Ferenci, Umesh Shukla, Ronald Kalmeijer, Oliver Lenz, Bart Fevery, Chris Corbett, Maria Beumont, Wolfgang Jessner

**Affiliations:** 1 New Zealand Liver Transplant Unit, Auckland City Hospital, Auckland, New Zealand; 2 Orlando Immunology Center, Orlando, FL USA; 3 Outpatients Clinic for Hepatology, ID Clinic, Myslowice, Poland; 4 Storr Liver Centre, Westmead Institute for Medical Research, Westmead Hospital and University of Sydney, New South Wales, Australia; 5 Hospital Quiron De Valencia, Valencia, Spain; 6 Reference and Training Center IST/AIDS – State Program of São Paulo, São Paulo, Brazil; 7 Academic Medical Center, Amsterdam, The Netherlands; 8 Institution of Russian Academy of Sciences Central Clinical Hospital, Moscow, Russia; 9 Leberstudienzentrum Kiel GbR, Kiel, Germany; 10 Campus Stuivenberg, Antwerpen, Belgium; 11 Allgemeines Krankenhaus der Stadt Wien, Universitätsklinik für Innere Medizin III, Wien, Austria; 12 Janssen Research & Development, LLC, Titusville, NJ USA; 13 Janssen Pharmaceutica NV, Beerse, Belgium

**Keywords:** Chronic hepatitis C, Direct-acting antiviral therapy, Peginterferon, Ribavirin, Safety, Simeprevir, Sustained virologic response

## Abstract

**Background:**

This Phase 3, open-label, rollover study (NCT01323244) investigated the efficacy and safety of simeprevir plus peginterferon α-2a (PegIFNα-2a) and ribavirin (RBV) in a well-characterized population of HCV genotype 1 (GT1)-infected treatment-experienced patients.

**Methods:**

Patients who had failed PegIFNα/RBV treatment in the placebo arm of a previous Phase 2/3 simeprevir study (Phase 2/3 group, *n* = 125), or had been exposed to HCV direct-acting antivirals (simeprevir or other) for up to 14 days in a selected Phase 1 study (Phase 1 group, *n* = 16), were eligible. Phase 2/3 group patients were classified according to prior relapse, breakthrough, or non-response (null response, partial response, non-classifiable non-response) to PegIFNα/RBV. Eight patients in the Phase 1 group received short-term (≤14 days) simeprevir.

Treatment comprised simeprevir 150 mg once daily (QD) plus PegIFNα-2a/RBV for 12 weeks followed by PegIFNα-2a/RBV for 12 or 36 weeks (using response-guided therapy [RGT] to determine total treatment duration in Phase 2/3 prior relapsers or breakthrough) or 36 weeks fixed (Phase 2/3 group non-responders and Phase 1 group). The primary endpoint was sustained virologic response 12 weeks after planned end of treatment (SVR12).

**Results:**

Phase 2/3 group: SVR12 rate was 69.6% (87/125) overall; 92.7% (51/55), 60.0% (6/10), 64.3% (18/28), and 36.7% (11/30) in patients with prior relapse, viral breakthrough, partial response, or null response, respectively. SVR12 rates were similar for patients with HCV GT1a (66.0% [33/50]) and GT1b infection (72.0% [54/75]) and among HCV GT1a-infected patients with/without a baseline Q80K polymorphism (66.7% [8/12] and 65.8% [25/38], respectively). The majority of RGT-eligible patients (prior viral relapse or breakthrough) met RGT criteria (89.2% [58/65]); of these, 89.7% (52/58) achieved SVR12. Overall, 16.0% (20/125) of patients experienced on-treatment failure and 14.4% (18/125) experienced post-treatment failure (15 relapses, 3 missing data). Phase 1 group (simeprevir-naïve and -experienced patients combined): SVR12 rate was 37.5% (6/16). Safety and tolerability findings were comparable to those of the feeder studies.

**Conclusions:**

The majority of RGT-eligible patients met criteria for shortening treatment to 24 weeks in total. Simeprevir 150 mg QD with PegIFNα-2a/RBV led to a high SVR rate among prior relapsers with HCV GT1 infection. No new safety signals were noted.

**Trial registration:**

NCT01323244. (date of registration: March 24, 2011).

**Electronic supplementary material:**

The online version of this article (doi:10.1186/s12879-017-2444-3) contains supplementary material, which is available to authorized users.

## Background

Until 2011, the standard of care for chronic hepatitis C virus (HCV) was peginterferon-α (PegIFNα) with ribavirin (RBV). This combination led to sustained virologic response (SVR) rates of 38–66% in treatment-naïve patients infected with HCV genotype 1 (GT1) [1, 2], 14% in patients who had relapsed after PegIFN-based therapy [3], and 9% in prior non-responders [3]. This suboptimal efficacy against HCV GT1 infection, together with long treatment durations (typically 48 weeks) and high rates of treatment discontinuations and dose reductions due to adverse events (AEs), made PegIFNα/RBV an unsatisfactory regimen [4, 5].

Direct-acting antiviral agents (DAAs) have significantly improved treatment options for chronic HCV infection; firstly, in combination with PegIFN and RBV and, more recently, as IFN-free DAA combination regimens. Simeprevir, an oral, once-daily (QD) HCV NS3/4A protease inhibitor with antiviral activity against HCV GT 1, 2, 4, 5, and 6 [6–9], is approved in combination with PegIFN and RBV for chronic HCV GT1 and GT4 infection with or without human immunodeficiency virus (HIV) co-infection in the USA and European Union (EU). Simeprevir is also approved as part of an IFN-free combination with sofosbuvir (a QD pangenotypic HCV nucleotide-analogue non-structural protein 5B [NS5B] polymerase inhibitor) for HCV GT1 infection in the USA, and GT1, GT4, and HCV/HIV co-infection in the EU [10, 11].

Prior to the availability of IFN-free therapies, in two Phase 2 studies, SVR rates of 81–86% and 67–80%, respectively, were achieved in treatment-naïve and treatment-experienced patients (prior non-responders, partial responders, and relapsers to therapy with PegIFN/RBV) with HCV GT1 infection treated with simeprevir plus PegIFNα/RBV (PILLAR [6] and ASPIRE [9]). In three Phase 3 studies in HCV GT1-infected patients, simeprevir for 12 weeks combined with 24 or 48 weeks of PegIFNα/RBV achieved significantly higher SVR rates compared with 48 weeks of PegIFNα/RBV (treatment naïve: 80% vs. 50% in QUEST-1 [12] and 81% vs. 50% in QUEST-2 [13]; prior PegIFN relapsers: 79% vs. 36% in PROMISE [14]). Furthermore, nearly 90% of patients met response-guided treatment (RGT) criteria and were eligible to shorten the duration of PegIFNα/RBV treatment to 24 weeks.

Here we report the results of a Phase 3 study that provided access to simeprevir in combination with PegIFNα-2a/RBV to HCV GT1-infected patients who had failed PegIFNα/RBV in the placebo group of one of the Phase 2/3 simeprevir studies mentioned above. In addition, a group of 16 patients who had been exposed to a DAA for up to 14 days in a Phase 1 study were included. This allowed evaluation of the efficacy and safety of simeprevir in a population that was well characterized in terms of prior response to PegIFN/RBV therapy. IFN-based treatment is still the standard of care in certain countries where IFN-free regimens are not yet available. The findings of this study may be of interest to healthcare professionals in such settings.

## Methods

### Patients

Patients with HCV GT1 infection who had failed PegIFNα/RBV in the placebo arm of a Phase 2/3 simeprevir study (Phase 2/3 group: did not achieve undetectable HCV RNA levels at the end of treatment [EOT] or had relapsed [confirmed detectable HCV RNA] within 1 year after EOT) **OR** had been exposed to a DAA for up to 14 days in a selected Phase 1 study (Phase 1 group) were enrolled. All patients were required to have completed the last study-related assessment in their previous study.

Patients in the Phase 2/3 group were recruited from two Phase 2 studies (ASPIRE [NCT00980330] [9] and PILLAR [NCT00882908] [6]) and from three Phase 3 studies (QUEST-1 [NCT01289782] [12], QUEST-2 [NCT01290679] [13], and PROMISE [NCT01281839] [14]).

Patients in the Phase 2/3 group were classified according to prior viral relapse, viral breakthrough, or non-response (i.e., null response, partial response, or non-classifiable non-response) based on their response to PegIFNα-2b/RBV in the feeder study.

Patients in the Phase 1 group were recruited from the TMC649128HPC1002 Phase 1 study (NCT01391117; data on file), in which patients had received an investigational nucleoside analogue NS5B inhibitor TMC649128 for 10 or 14 days, and from the TMC647055HPC1001 study (NCT01202825 [Cohort 8]), in which patients received the combination of simeprevir with an investigational non-nucleoside analogue NS5B inhibitor TMC647055 for 10 days [15].

Key exclusion criteria included: hepatic decompensation, newly diagnosed liver disease of non-HCV etiology, hepatocellular carcinoma, co-infection with HCV non-GT1, HIV, or hepatitis B virus (hepatitis B surface antigen positive), any medical condition that contraindicated the use of PegIFNα or RBV, history of malignancy within 5 years of the screening visit, and previous treatment with any HCV DAA therapy, other than short-term (≤14 days) DAA treatment in the Phase 1 study. All patients provided written informed consent.

### Study design

This was a Phase 3, open-label, multicenter study (ClinicalTrials.gov number: NCT01323244). The study protocol and amendment(s) were reviewed by the Independent Ethics Committees and Institutional Review Boards at each study center. The study was conducted in accordance with the Declaration of Helsinki, and was consistent with Good Clinical Practice and applicable regulatory requirements.

**Fig. 1:**
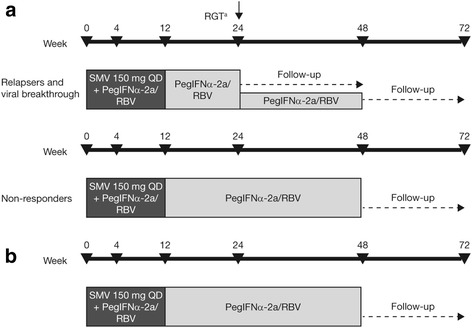
Study designs for (**a**) the Phase 2/3 group and (**b**) the Phase 1 group. ^a^ As part of a response-guided treatment duration, all HCV therapy was stopped at Week 24 in patients who achieved HCV RNA levels <25 IU/ml (detectable or undetectable) at Week 4, and <25 IU/ml undetectable HCV RNA levels at Week 12. For patients who did not achieve these criteria, PegIFNα-2a and RBV treatment continued until Week 48. *PegIFNα-2a/RBV* peginterferon α-2a with ribavirin, *QD* once daily, *RGT* response-guided therapy, *SMV* simeprevir

The study comprised a screening period (≤6 weeks), a 24- or 48-week treatment period, and a follow-up period of 24 weeks after the planned EOT. Patients in the Phase 2/3 group with previous relapse or breakthrough received simeprevir (150 mg QD, orally) for 12 weeks in combination with PegIFNα-2a (180 μg/week, subcutaneously) and RBV (1000 mg/day or 1200 mg/day, orally, depending on body weight [<75 kg or ≥75 kg, respectively]) followed by PegIFNα-2a/RBV for 12 or 36 weeks (Fig. 1a). The criteria for RGT were modified during the course of the study. For patients enrolled under the initial study protocol or an early amendment, treatment was stopped at Week 24 when HCV RNA was <25 IU/ml (detectable or undetectable) at Week 4 and <25 IU/ml undetectable at Week 12. For patients enrolled under a later amendment, treatment was stopped at Week 24 if HCV RNA was <25 IU/ml (undetectable) at Week 4.

Patients in the Phase 2/3 group who were classified as non-responders, and those in the Phase 1 group, received simeprevir 150 mg plus PegIFNα-2a/RBV for 12 weeks, followed by 36 weeks of PegIFNα-2a/RBV (Fig. 1b).

Dose adjustments or treatment interruptions were permitted for PegIFNα-2a/RBV in response to AEs or tolerability issues; no dose adjustments of simeprevir were permitted. A single interruption (≤4 days) in simeprevir treatment was allowed in response to AEs.

In accordance with the virologic stopping rules, all study medication was discontinued if HCV RNA plasma level was >1000 IU/ml at Week 4 or 12, or when HCV RNA was confirmed detectable at Week 24 or 36 (initial study protocol). Patients enrolled under a later protocol amendment permanently discontinued all study medication when HCV RNA was ≥25 IU/ml at Week 4 or when HCV RNA was confirmed detectable at Week 12, 24, or 36.

### Study endpoints

The primary efficacy endpoint was SVR12 (defined as HCV RNA <25 IU/ml undetectable at the actual EOT and HCV RNA <25 IU/ml detectable or undetectable 12 weeks after the planned EOT).

Secondary endpoints included SVR 24 weeks after the planned EOT (SVR24); undetectable HCV RNA (<25 IU/ml undetectable) and HCV RNA <25 IU/ml detectable at Weeks 4, 12, 24, 36, and 48; on-treatment failure (HCV RNA <25 IU/ml detectable or ≥25 IU/ml at EOT); viral breakthrough (>1 log_10_ IU/ml increase in HCV RNA from the lowest level reached, or HCV RNA >100 IU/ml when previously <25 IU/ml); viral relapse (undetectable HCV RNA at EOT and HCV RNA ≥25 IU/ml during follow-up); normalized alanine aminotransferase (ALT) at EOT and SVR time points; and AEs and laboratory abnormalities.

### Assessments

Blood samples for the determination of HCV RNA were taken at screening, Days 1, 7, 14, and 28, every 4 weeks thereafter until Week 24, at Weeks 36 and 48 for those continuing treatment until Week 48, and at Weeks 4, 12, and 24 during follow-up. In patients who discontinued study medication early, HCV RNA measurements were obtained at the time of withdrawal, 4 weeks after withdrawal, and every 12 weeks until Week 72. HCV RNA was measured using the Roche COBAS® TaqMan® HCV assay (version 2.0 for use with the High Pure System, Pleasanton, CA, USA; lower limit of quantification 25 IU/ml and limit of detection 15 IU/ml).

Standard population sequencing to assess for simeprevir resistance-associated substitutions in the HCV NS3/4A protease domain was performed at baseline for all patients and post-baseline for patients not achieving SVR. If the interleukin-28b (*IL28B;* single nucleotide polymorphism rs12979860) status of the patient was unknown at the start of the study, *IL28B* genotyping was performed on blood samples obtained at screening.

Liver biopsy was required within 3 years prior to screening unless the patient had a contraindication to biopsy. In such cases, noninvasive assessment with Fibroscan or MR-Elastography was permitted. If a liver biopsy, or alternative non-invasive assessment, was not available prior to screening, the liver biopsy, or alternative non-invasive assessment had to be performed during the screening period.

Safety evaluations included AE reporting, clinical laboratory tests, vital sign measurements, and physical examinations. Laboratory abnormalities were classified by severity according to the World Health Organization grading scale (Grade 1: Mild; Grade 2: Moderate; Grade 3: Severe; Grade 4: Potentially life threatening) [16].

### Statistical analysis

Statistical analyses were performed using SAS® version 9.2(SAS Institute Inc., Cary, NC, USA). All analyses were performed on the intent-to-treat (ITT) population (all patients who received at least one dose of simeprevir). No formal sample size determination was performed. It was calculated that the placebo groups in the previous Phase 2/3 studies could potentially provide 539 patients. Assuming that 50% of patients from the Phase 2/3 studies would fail treatment for virologic reasons, it was estimated that 270 patients would roll over into the current study. With this sample size, and an expected response rate of 50%, the width of the 95% confidence interval (CI) around the anticipated response was 11.9%. For a sample size of 135 patients, the width of the 95% CI was 16.9%. HCV GT1-infected patients who had received short-term (≤14 days) DAA therapy in the Phase 1 study were also considered for inclusion.

Data from the Phase 2/3 group were analyzed overall and by subgroup according to treatment response in the feeder study. Safety and tolerability were evaluated for the overall population.

The 95% CI was calculated for the proportion of patients achieving SVR12 based on the normal approximation. In addition, a logistic regression model was used to determine the proportion of patients achieving SVR12 and 95% CI in the Phase 2/3 group. This model was adjusted for baseline HCV RNA level, *IL28B* genotype (CC, CT, TT), HCV geno/subtype (1a/other, 1b) and response to prior PegIFN/RBV treatment.

For all other response parameters in the Phase 2/3 group, 95% CIs were constructed around the observed values and the same logistic regression model as applied for the primary efficacy parameter was used. The time to achieve undetectable HCV RNA was estimated using Kaplan-Meier plots. Descriptive statistics were calculated for the change in log_10_ HCV RNA levels from baseline at all time points.

For the Phase 2/3 group, subgroup analyses were performed based on baseline HCV RNA levels, early viral response criteria, and baseline characteristics of race and age to investigate the possible effect on SVR12. In addition, a multivariate model applying cubic spline functions for continuous factors was fitted on the SVR12 data exploring the combined effect of several factors. Subgroup analyses were also performed based on the *IL28B* genotype and response in the feeder study.

Due to the limited number of patients in the Phase 1 group, data were assessed descriptively.

## Results

### Patients

The study was conducted at 75 centers in 22 countries between December 5, 2011 and March 31, 2015. Baseline demographic and clinical characteristics are summarized in Table 1. In both groups, the majority of patients were male (80/125 [64.0%] and 13/16 [81.3%] in the Phase 2/3 and Phase 1 groups, respectively) and the median age was 52.0 years (51.0 [range: 22–72] and 52.5 [range: 33–65] years in the Phase 2/3 and Phase 1 groups, respectively). Phase 2/3 and Phase 1 groups were combined for the safety analysis.

**Table 1:** Baseline demographics and clinical characteristics

Characteristic	Phase 2/3 group (*N* = 125)	Phase 1 group (*N* = 16)
Male, *n (%)*	80 (64.0)	13 (81.3)
Age (years), median (range)	51.0 (22–72)	52.5 (33–65)
BMI (kg/m^2^), median (range)	27.4 (18.9–45.8)	27.1 (20.4–35.3)
Race, *n (%)*		
White	121 (96.8)	14 (87.5)
Black/African American	3 (2.4)	1 (6.3)
American Indian/Alaskan native	0	1 (6.3)
Native Hawaiian or other Pacific Islander	1 (0.8)	0
Ethnicity, *n (%)*		
Hispanic or Latino	7 (5.6)	1 (6.3)
Not Hispanic or Latino	118 (94.4)	15 (93.8)
Time since diagnosis (years), median (range)	7.1 (2.1–35.4)	6.1 (0.7–20.4)
SMV therapy-experienced, yes, *n (%)*	125 (100)	8 (50)
PegIFN/RBV therapy-experienced, yes, *n (%)*	125 (100)	9 (56.2)
Response to last course of PegIFN/RBV therapy, *n (%)*		
Viral relapser	55 (44.0)	–
Viral breakthrough	10 (8.0)	–
Non-responder	60 (48.0)	–
Partial responder	28 (22.4)	–
Null responder	30 (24.0)	–
Other (non-classifiable non-responder)	2 (1.6)	–
Baseline HCV RNA (log_10_ IU/ml), median (range)	6.53 (4.9–7.8)	6.68 (5.6–7.2)
*IL28B* genotype, *n (%)*		
CC	20 (16.0)	1 (6.3)
CT	87 (69.6)	9 (56.3)
TT	18 (14.4)	6 (37.5)
HCV geno/subtype		
1a	50 (40.0)	14 (87.5)
1a with Q80K	12/50 (24.0)	3/14 (21.4)
1a without Q80K	38/50 (76.0)	11/14 (78.6)
1b	75 (60.0)	2 (12.5)
HOMA-IR,^a^ *n (%)*		
<2	46/120 (38.3)	4/14 (28.6)
≥2 to ≤4	43/120 (35.8)	7/14 (50.0)
>4	31/120 (25.8)	3/14 (21.4)
METAVIR fibrosis score, *n (%)*		
F0, 1, or 2	85 (68.0)	13 (81.3)
F3	18 (14.4)	3 (18.8%)
F4	22 (17.6)	0
Baseline ALT WHO toxicity grade, *n (%)*		
Grade 0	66 (52.8)	10 (62.5)
Grade 1/2	56 (44.8)	6 (37.5)
Grade 3	3 (2.4)	0
Grade 4	0	0

^a^ *N* = 120 (Phase 2/3 group), *N* = 14 (Phase 1 group)

*ALT* alanine aminotransferase, *BMI* body mass index, *HCV* hepatitis C virus, *HOMA-IR* homeostatic model assessment of insulin resistance, *IL28B* interleukin-28b, *PegIFN/RBV* peginterferon with ribavirin, *SMV* simeprevir, *WHO* World Health Organization

#### Phase 2/3 group

In total, 125 patients in the Phase 2/3 group received simeprevir treatment in the current study and represented the ITT population. The study was completed by 92.0% (115/125) of patients; the main reasons for study discontinuation were withdrawal of consent and lost to follow-up (both 4.0%). Thirteen patients discontinued simeprevir early (2.4% [3/125] due to AEs [including 2 patients who also stopped RBV and PegIFNα-2a due to an AE], 1.6% [2/125] due to withdrawal of consent, and 6.4% [8/125] due to reaching a virologic endpoint). In total, 79.2% (99/125) completed treatment with PegIFNα-2a and/or RBV (24 or 48 weeks). Median simeprevir treatment duration was 12.0 weeks. Median PegIFNα-2a/RBV treatment duration was 24.1 weeks.

Categorization according to prior treatment response was as follows: viral relapsers (44.0%; 55/125), viral breakthrough (8.0%; 10/125), or non-responder (48.0% [60/125]: null response, 24.0% [30/125]; partial response, 22.4% [28/125]; non-classifiable non-responder, 1.6% [2/125]).

The proportion of patients with baseline NS3 polymorphisms was 29.6% [37/125]. NS3 polymorphism Q80K was observed in 24.0% (12/50) of HCV GT1a-infected patients. None of the HCV GT1b-infected patients had a Q80K polymorphism. NS3 polymorphisms reducing simeprevir activity in vitro *–* other than the Q80K polymorphism – were observed in 1 patient carrying D168E at baseline.

#### Phase 1 group

Sixteen patients in the Phase 1 group received simeprevir treatment in the current study and represented the Phase 1 ITT population. The study was completed by 75.0% (12/16) of patients; the main reasons for study discontinuation were withdrawal of consent (18.8%) and lost to follow-up (6.3%). One patient discontinued simeprevir early due to withdrawal of consent (6.3% [1/16]). In total, 56.3% (9/16) of patients in the Phase 1 group completed treatment with PegIFNα-2a and/or RBV (24 or 48 weeks). Median simeprevir treatment duration was 12.1 weeks. Median PegIFNα-2a/RBV treatment duration was 48.1 weeks.

Fifty-percent of patients (8/16) were simeprevir treatment-naïve and 50.0% (8/16) were simeprevir treatment-experienced. Of the 8 patients who had not been previously exposed to simeprevir in a Phase 1 study, 6 were prior non-responders to PegIFN/RBV treatment, 1 was a prior relapser to PegIFN/RBV, and 1 was treatment-naïve.

The proportion of patients with baseline NS3 polymorphisms was 50.0% (8/16). NS3 polymorphism Q80K was observed in 21.4% (3/14) of HCV GT1a-infected patients. None of the HCV GT1b-infected patients had a Q80K polymorphism. Among the 8 patients in the Phase 1 group with prior short-term exposure to simeprevir, 6 had an emerging R155K mutation that was still detected in 2 patients at baseline in the current study.

### Efficacy

#### Phase 2/3 group

##### Sustained virologic response

The SVR12 rate was 69.6% (87/125; 95% CI 61.5–77.7). When patients were classified according to prior PegIFN/RBV response, the highest SVR12 rate was seen for prior relapsers (92.7% [51/55]). The SVR12 rate for prior viral breakthrough patients was 60.0% (6/10). For prior non-responders – including partial responders, null responders, and non-classifiable non-responders – SVR12 rates were 64.3% (18/28), 36.7% (11/30), and 50.0% (1/2), respectively. In the Phase 2/3 group, RGT criteria were met by the majority (89.2% [58/65]) of RGT-eligible patients. Of these, 89.7% (52/58) achieved SVR12.

All patients achieving SVR12 also achieved SVR24; 94.6% (87/92) of patients achieving SVR4 achieved SVR12. Considering both groups of prior non-responder patients together (i.e., patients with prior relapse [RGT eligible] and prior non responders [non RGT eligible), the SVR12 rate was 81.1% (73/90) in patients with rapid virologic response (RVR, HCV RNA <25 IU/ml undetectable at Week 4).

SVR12 rates according to baseline characteristics are summarized in Table 2. SVR12 rates were similar for patients with HCV GT1a (66.0% [33/50]) and HCV GT1b (72.0% [54/75]) in the Phase 2/3 group. HCV GT1a-infected patients with and without a baseline Q80K polymorphism had SVR12 rates of 66.7% (8/12) and 65.8% (25/38), respectively. The HCV GT1b-infected patient with a D168E at baseline achieved SVR12.

**Table 2:** SVR12 according to patient baseline characteristics (ITT population)

*n/N (%)*	Phase 2/3 group (*N* = 125)	Phase 1 group (*N* = 16)^a^
HCV geno/subtype		
1a	33/50 (66.0)	5/14 (35.7)
with baseline Q80K	8/12 (66.7)	2/3 (66.7)
without baseline Q80K	25/38 (65.8)	3/11 (27.3)
1b	54/75 (72.0)	1/2 (50.0)
*IL28B*		
CC	17/20 (85.0)	1/1 (100.0)
CT	58/87 (66.7)	3/9 (33.3)
TT	12/18 (66.7)	2/6 (33.3)
HOMA-IR		
<2	39/46 (84.8)	2/4 (50.0)
≥2 to ≤4	25/43 (58.1)	3/7 (42.9)
>4	20/31 (64.5)	0/3
METAVIR fibrosis score		
F0–F2	64/85 (75.3)	5/13 (38.5)
F3–F4	23/40 (57.5)	1/3 (33.3)
F3	11/18 (61.1)	1/3 (33.3)
F4	12/22 (54.5)	0

^a^Due to small patient numbers, these data should be interpreted with caution

*HCV* hepatitis C virus, *HOMA-IR* homeostatic model assessment of insulin resistance, *IL28B* interleukin-28b, *ITT* intent-to-treat, *SVR12* sustained virologic response 12 weeks after planned end of treatment

In the multivariate analysis that considered all factors, baseline homeostatic model assessment of insulin resistance (HOMA-IR), response to prior PegIFN/RBV treatment, complete early virologic response (<25 IU/ml undetectable at Week 12), and HCV RNA <25 IU/ml at Week 4 were the characteristics identified as having a statistically significant effect on the probability of achieving SVR12 (see Additional file 1: Table S1).

Overall, and as expected, the SVR12 rate was higher for patients with *IL28B* genotype CC (vs. CT or TT), METAVIR F0–F2 (vs. F3–F4), and for patients with HOMA-IR <2 (vs. HOMA-IR ≥2 to ≤4 or >4) (Table 2).

##### On-treatment virologic response

During the first 4 weeks of treatment, an initial rapid reduction in plasma HCV RNA was evident (Table 3). RVR was achieved in 72.0% (90/125) of patients.

**Table 3:** On-treatment virologic response for all patients (ITT population)

*n/N (%)*	Phase 2/3 group (*N* = 125)	Phase 1 group (*N* = 16)
Week 2		
<25 IU/ml undetectable	30/125 (24.0)	1/15 (6.7)
<25 IU/ml undetectable/detectable	99/125 (79.2)	5/15 (33.3)
Week 4		
<25 IU/ml undetectable (RVR)	90/125 (72.0)	7/15 (46.7)
<25 IU/ml undetectable/detectable	112/125 (89.6)	15/15 (100.0)
Week 12		
<25 IU/ml undetectable (cEVR)	108/114 (94.7)	13/15 (86.7)
<25 IU/ml undetectable/detectable	113/114 (99.1)	14/15 (93.3)
Week 24		
<25 IU/ml undetectable	102/105 (97.1)	12/13 (92.3)
<25 IU/ml undetectable/detectable	104/105 (99.0)	13/13 (100.0)
Week 36		
<25 IU/ml undetectable	38/44 (86.4)	10/11 (90.9)
<25 IU/ml undetectable/detectable	41/44 (93.2)	11/11 (100.0)
Week 48		
<25 IU/ml undetectable	36/39 (92.3)	9/9 (100.0)
<25 IU/ml undetectable/detectable	37/39 (94.9)	9/9 (100.0)
EOT		
<25 IU/ml undetectable (EOTR)	105/125 (84.0)	13/15 (86.7)
<25 IU/ml undetectable/detectable	109/125 (87.2)	14/15 (93.3)

*cEVR* complete early virologic response, *EOTR* end of treatment response, *ITT* intent-to-treat, *RVR* rapid virologic response

##### Virologic breakthrough, viral relapse, or treatment failure

Viral breakthrough was noted in 9.6% (12/125) of patients in the Phase 2/3 group (of which 10/12 patients had been PegIFN/RBV null responders in the feeder study). None of the patients with viral breakthrough achieved SVR12. All but 1 of the patients with viral breakthrough met a virologic stopping rule; these comprised 7 patients at Week 4, and 4 patients at Weeks 12, 24, or 36. The proportion of patients with on-treatment failure was 16.0% (20/125) (Table 4).

**Table 4:** On-treatment and post-treatment failure by trial phase (ITT population)

*n/N (%)*	Phase 2/3 group (*N* = 125)	Phase 1 group (*N* = 16)
Failure	38/125 (30.4)	10/16 (62.5)
On-treatment failure	20/125 (16.0)	3/16 (18.8)
Met stopping rule at Week 4/12/24/36	13/125 (10.4)	0/16
Other (detectable at EOT)	5/125 (4.0)	2/16 (12.5)
Viral breakthrough	2/125 (1.6)	1/16 (6.3)
Completed PegIFNα-2a and/or RBV	3/125 (2.4)	0/16
Met stopping rule at Week 4/12/24/36	1/125 (0.8)	0/16
Other (detectable at EOT)	1/125 (0.8)	0/16
Viral breakthrough	1/125 (0.8)	0/16
Discontinued PegIFNα-2a and RBV	17/125 (13.6)	3/16 (18.8)
Met stopping rule at Week 4/12/24/36	12/125 (9.6)	0/16
Other (detectable at EOT)	4/125 (3.2)	2/16 (12.5)
Viral breakthrough	1/125 (0.8)	1/16 (6.3)
Post-treatment failure	18/125 (14.4)	7/16 (43.8)
Viral relapse	15/125 (12.0)	5/16 (31.3)
Discontinued PegIFNα-2a and RBV	3/125 (2.4)	3/16 (18.8)
Completed PegIFNα-2a and/or RBV	12/125 (9.6)	2/16 (12.5)
Missing at time point of SVR12	3/125 (2.4)	2/16 (12.5)

*EOT* end of treatment, *ITT* intent-to-treat, *PegIFNα-2a/RBV* peginterferon α-2a with ribavirin, *SVR12* sustained virologic response 12 weeks after planned end of treatment

Post-treatment failure was observed in 14.4% (18/125) of patients, with the majority of patients experiencing viral relapse (12.0% [15/125]). Missing data at the SVR12 assessment time point was the reason for post-treatment failure in 2.4% (3/125) patients. Fifteen of 104 patients (14.4%) with undetectable HCV RNA at EOT had a viral relapse. In all patients with virologic failure, available sequencing data did not suggest any cases of reinfection.

Twenty-seven of 33 patients (81.8%) with treatment failure and available sequencing data had emerging substitutions at NS3 amino acid positions 80, 122, 155, and/or 168 at the time of failure (Table 5).

**Table 5:** Patients with emerging mutations^a^ in patients with failure,^b^ by baseline Q80K polymorphism (ITT population)

*n/N (%)*	Phase 2/3 group (*N* = 125)	Phase 1 group (*N* = 16)
Failure	38	10
Sequencing data		
Available^c^	33/38	8/10
Not available	5/38	2/10
Emerging mutations at failure	27/33 (81.8)	7/8 (87.5)
Without Q80K at baseline	24/33 (72.7)	7/8 (87.5)
D168V	9/33 (27.3)	4/8 (50.0)
Q80L + R155K	2/33 (6.1)	0/8
D168E/V + F169I	1/33 (3.0)	0/8
Q80L + D168V	1/33 (3.0)	0/8
Q80L + I132L + R155K	1/33 (3.0)	0/8
Q80R	1/33 (3.0)	0/8
Q80R + D168E	1/33 (3.0)	0/8
Q80R + D168E/V	1/33 (3.0)	0/8
Q80R + R155K	1/33 (3.0)	0/8
R155K	1/33 (3.0)	0/8
R155K + D168A/N/T	1/33 (3.0)	0/8
R155K + D168E/V + I170V	1/33 (3.0)	0/8
R155K + N174 K	1/33 (3.0)	0/8
R155K + N174S	1/33 (3.0)	0/8
R155Q + D168V	1/33 (3.0)	0/8
D168A/V + I170V	0/33	1/8 (12.5)
Q80K	0/33	1/8 (12.5)
S122R + R155K	0/33	1/8 (12.5)
With Q80K at baseline	3/33 (9.1)	0/8
R155K	2/33 (6.1)	0/8
D168E	1/33 (3.0)	0/8
No emerging mutation at failure	6/33 (18.2)	1/8 (12.5)
Without Q80K at baseline	6/33 (18.2)	0/8
With Q80K at baseline	0/33	1/8 (12.5)

^a^Considering NS3 positions 36, 41, 43, 54, 55, 80, 107, 122, 132, 138, 155, 156, 158, 168, 169, 170, 174, and 175

^b^Failures: all patients with failure

^c^Only patients with baseline and post-baseline sequencing data are considered

*ITT* intent-to-treat

##### Normalization of ALT levels

The majority of patients (71.2% [42/59]) had normalization of their ALT during treatment. Median time to normalization of ALT was 8.0 (95% CI 2.0–24.1) weeks.

#### Phase 1 group

##### Sustained virologic response

**Fig. 2:**
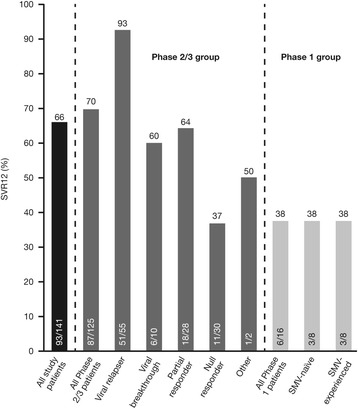
SVR12 for all patients (ITT population) and by response to prior treatment. *ITT* intent-to-treat, *SMV* simeprevir, *SVR12* sustained virologic response 12 weeks after planned end of treatment

The SVR12 rate was 37.5% (6/16; 95% CI 13.8–61.2), and no difference in SVR12 rate was observed between simeprevir-naïve and experienced patients (both 3/8 [37.5%]) [Fig. 2]. All patients who achieved SVR4 (37.5% [6/16]) also achieved SVR12 and SVR24. Among patients with RVR, the SVR12 rate was 42.9% (3/7).

Two of the 3 HCV GT1a-infected patients with Q80K polymorphism at baseline achieved SVR12 (Table 2). One of the 2 HCV GT1a-infected patients with an R155K at baseline achieved SVR12. Of note, the R155K was found emerging in both patients during previous short-term exposure to simeprevir in the Phase 1 study, and was still present at baseline of the current study.

The SVR12 rate was higher for patients with *IL28B* genotype CC (vs. CT or TT), METAVIR F0–F2 (vs. F3–F4), and for patients with HOMA-IR <2 (vs. HOMA-IR ≥2 to ≤4 or >4) (Table 2).

##### On-treatment virologic response

During the first 4 weeks of treatment, an initial rapid reduction in plasma HCV RNA was evident (Table 3). RVR was achieved in 7/15 (46.7%) patients.

##### Virologic breakthrough, viral relapse, or treatment failure

Three patients experienced on-treatment failure (3/16 [18.8%]), none of whom achieved SVR12 (Table 4). One of 15 patients (6.7%) experienced viral breakthrough and did not achieve SVR12; this patient met the virologic stopping rule at Week 12.

Post-treatment failure was observed in 7/16 (43.8%) patients, with the majority of patients experiencing viral relapse (5/16 [31.3%]). Missing data at the SVR12 assessment time point was the reason for post-treatment failure in 2/16 (12.5%) of patients. Five of 12 patients (41.7%) with undetectable HCV RNA at EOT had a viral relapse. In all patients with virologic failure, available sequencing data did not suggest any cases of reinfection.

Seven of 8 patients (87.5%) with treatment failure and sequencing data available had emerging substitutions at NS3 amino acid positions 80, 122, 155, and/or 168 at the time of failure (Table 5).

##### Normalization of ALT levels

The majority of patients (5/6 [83.3%]) had normalization of their ALT during treatment. Median time to normalization of ALT was 24.1 (95% CI 2.1–36.1) weeks.

### Safety

The majority (90.8%) of patients experienced at least one AE during the simeprevir plus PegIFNα-2a/RBV treatment phase. The most common AEs (>20% of patients) were fatigue (32.6%), neutropenia (22.7%), and influenza-like illness (22.0%). The majority of AEs were Grade 1 or 2 in severity; Grade 3 or 4 AEs were reported in 29.1% of patients and were considered at least possibly related to simeprevir in 6.4% of patients (Table 6). The most frequent Grade 3/4 AE (>5% of patients) was neutropenia (14.2%). AEs leading to permanent discontinuation of study drugs were uncommon. Three (2.1%) patients permanently discontinued simeprevir due to an AE (Grade 3 hyperbilirubinemia, panic attack, Grade 3 photosensitivity reaction; 1 patient each). The panic attack and photosensitivity reaction AEs also led to permanent discontinuation of RBV and PegIFNα-2a. Serious AEs (SAEs) were reported in 5 patients (3.5%: gastritis and bronchitis [both Grade 2], pancytopenia, hand fracture, and renal cell carcinoma [all Grade 3]). None of the SAEs were reported in more than 1 patient and none were considered possibly related to study medication, with the exception of pancytopenia, which was considered very likely related to RBV and possibly related to PegIFNα-2a. No deaths were reported.

**Table 6:** Summary of AEs (ITT population)

	SMV + PegIFNα-2a/RBV treatment phase	Entire treatment phase
*n (%)*	(*N* = 141)	(*N* = 141)
Any AE	128 (90.8)	131 (92.9)
Worst Grade 1 or 2	87 (61.7)	81 (57.4)
Worst Grade 3	36 (25.5)	45 (31.9)
Worst Grade 4	5 (3.5)	5 (3.5)
Worst Grade 3/4, at least possibly related to SMV	9 (6.4)	9 (6.4)
Treatment-related AE	127 (90.1)	131 (92.9)
At least possibly related to SMV	93 (66.0)	93 (66.0)
At least possibly related to RBV	99 (70.2)	109 (77.3)
At least possibly related to PegIFNα-2a	123 (87.2)	126 (89.4)
Any AE with fatal outcome	0	0
Any SAE	5 (3.5)	9 (6.4)
At least possibly related to SMV	0	0
AE leading to permanent stop of at least one drug	6 (4.3)	9 (6.4)
SMV^a^	3 (2.1)	3 (2.1)
PegIFNα-2a and/or RBV	3 (2.1)	6 (4.3)
Events of special interest	17 (12.1)	17 (12.1)
Increased bilirubin	17 (12.1)	17 (12.1)
Events of clinical interest	78 (55.3)	87 (61.7)
Rash (any type)^b^	31 (22.0)	36 (25.5)
Pruritus	25 (17.7)	26 (18.4)
Photosensitivity conditions	8 (5.7)	8 (5.7)
Neutropenia	33 (23.4)	38 (27.0)
Anemia	11 (7.8)	23 (16.3)
Dyspnea	15 (10.6)	17 (12.1)

^a^Without regard to PegIFNα-2a and RBV

^b^Includes photosensitivity conditions

*AE* adverse event; *ITT* intent-to-treat, *PegIFNα-2a/RBV* peginterferon α-2a with ribavirin, *SAE* serious adverse event, *SMV* simeprevir

There were no unexpected observations in terms of the frequency or severity of AEs of special/clinical interest, including rash (any type), pruritus, anemia, photosensitivity conditions, neutropenia, and dyspnea (Table 6). The majority of the AEs of special/clinical interest were Grade 1/2 in severity. Grade 3 and Grade 4 neutropenia AEs were reported in 11.3% and 3.5% of patients, respectively. Other Grade 3 AEs of special/clinical interest were reported in ≤3.5% of patients and there were no other Grade 4 AEs of special/clinical interest.

None of the AEs of special/clinical interest were serious and only a small number resulted in permanent discontinuation of study treatment: Grade 3 hyperbilirubinemia (increased bilirubin AE) led to permanent discontinuation of simeprevir in 1 patient (0.7%) (protocol-driven) and a Grade 3 photosensitivity reaction (rash AE, which was also a photosensitivity condition) led to permanent discontinuation of simeprevir, PegIFNα-2a, and RBV in 1 patient (0.7%; mentioned previously). The patient with Grade 3 hyperbilirubinemia, considered probably related to simeprevir, also experienced Grade 1 rash, Grade 3 pruritus, Grade 2 ALT, and Grade 1 aspartate aminotransferase (AST) AEs. The Grade 3 photosensitivity reaction which led to discontinuation of simeprevir, PegIFNα-2a, and RBV was considered to be very likely related to simeprevir and was subsequently reported as resolved following treatment discontinuation.

The most frequently noted laboratory parameter abnormalities (>25% of patients) during the simeprevir plus PegIFNα-2a/RBV treatment phase for which Grade ≥3 was noted included decreases in neutrophil and precursor count (79.3%, any grade), hyperbilirubinemia (53.6%, any grade), and decreases in platelet count (25.7%, any grade). Increases in direct and indirect bilirubin levels above normal limits (any grade) were reported in 46.4% and 30.7% of patients, respectively. Decreases in neutrophil and precursor count were the only graded treatment-emergent laboratory abnormalities for which Grade 3 or 4 abnormalities were observed in >10% of patients (19.3%). Grade 3 hyperbilirubinemia was observed in 9 (6.4%) patients and Grade 4 hyperbilirubinemia in 1 (0.7%) patient.

Grade 3 ALT or AST AEs were experienced by 2.1% (3/141) of patients during the simeprevir plus PegIFNα-2a/RBV treatment phase (ALT, 2 patients; AST, 1 patient). Grade 3 ALT and AST events were not considered to be related to the study drug. No Grade 4 ALT/AST AEs were reported during treatment with simeprevir.

## Discussion

This open-label study was designed to provide access to simeprevir treatment in combination with PegIFNα-2a and RBV for a well characterized treatment-experienced patient population with HCV GT1 infection in terms of baseline characteristics and prior response to PegIFN/RBV, after they had completed participation in a prior study and at a time when current therapeutic IFN-free alternatives were not yet available.

The Phase 2/3 group comprised a high proportion of prior relapsers (44.0%); null responders and partial responders (each approximately 25%) to PegIFNα/RBV. Fewer than 10% of patients had experienced viral breakthrough. The SVR12 rate (primary efficacy endpoint) was 69.6% (87/125); all patients who achieved SVR12 also went on to achieve SVR24. The majority (89.2%) of patients eligible for RGT met RGT criteria and received a shorter 24-week duration of PegIFNα-2a/RBV therapy. The SVR12 rate in this group of patients was 89.7%. There was a small difference in the number of patients (50/51 [98.0%]) who qualified for shorter treatment duration using the original RGT as compared to those who qualified for shorter treatment duration with the modified criteria in the same group of patients (45/51 [88.2%]) retrospectively. SVR12 was achieved in 45 of 50 (90%) patients who qualified for shorter treatment duration with the original RGT versus 41 of the 45 (91.1%) patients from the same group who qualified for shorter treatment with the modified RGT retrospectively.

The virologic response for non-responders in the Phase 2/3 group (50%) was comparable to the overall SVR12 rate reported with simeprevir plus PegIFNα-2a/RBV (54%) and telaprevir plus PegIFNα-2a/RBV (55%) in a previous non-responder HCV GT1-infected population in the ATTAIN study [17].

While the HCV geno/subtype influences response to HCV therapy and higher rates of virologic failure are seen in GT1a-infected patients compared with GT1b [18], the SVR12 rates in HCV GT1a- and GT1b-infected patients in the Phase 2/3 group of the present study were comparable (66.0% and 72.0%, respectively).

Phase 3 studies evaluating the combination of simeprevir with PegIFN/RBV indicated lower SVR rates in HCV GT1a-infected patients with a Q80K baseline polymorphism compared to those without Q80K polymorphisms [12–14]. In the Phase 2/3 group of this study, no difference in SVR12 rates was observed among GT1a patients with and without a Q80K polymorphism (66.7% and 65.8%, respectively).

The proportion of patients with on-treatment failure in the Phase 2/3 group was 16.0%. The incidence of viral breakthrough was <10% and 10/12 patients with viral breakthrough were prior null responders to PegIFN/RBV treatment. Consistent with the simeprevir resistance profile observed in HCV GT1-infected patients who failed simeprevir plus PegIFNα/RBV in Phase 2/3 studies [19], the majority of patients with failure had emerging mutations at NS3 amino acid positions 80, 122, 155, and/or 168 at the time of failure. The impact of these treatment-emergent NS3 mutations on retreatment options is not clear. Although they represent the dominant viral population at the time of virologic failure, these NS3 mutations are rapidly replaced by fitter wild type viruses. In half of patients with failure, and treatment-emergent NS3 mutations at time of failure, in Phase 2/3 simeprevir/PegIFN studies, these mutations were no longer detected at the end of the studies after a median follow-up time of 28 weeks [19].

This observation raises the possibility that patients who fail simeprevir plus PegIFNα/RBV could be retreated with a simeprevir-containing regimen. However, the rapid development of IFN-free combination DAA regimens has provided more effective retreatment options, which are recommended by current treatment guidelines [18, 20].

Consistent with the established association between improved response (SVR) to PegIFN/RBV and presence of the *IL28B* rs12979860 CC genotype [21, 22], a higher SVR12 rate was seen in patients with CC genotype (85.0%) compared with the CT (66.7%) and TT (66.7%) genotypes in the Phase 2/3 group. SVR12 rates were also somewhat higher among patients with less severe fibrosis and in patients with HOMA-IR <2. The latter finding supports previous reports which suggest an association between lower virologic cure rates and elevated HOMA-IR, a surrogate marker of insulin resistance [23].

The tolerability profile of simeprevir in the current study was comparable to that reported in the previous Phase 2/3 studies investigating simeprevir in combination with PegIFNα-2a/RBV in patients with HCV GT1 infection [6, 9, 12–14]. Fatigue (32.6%), neutropenia (22.7%), and influenza-like illness (22.0%) were the most common AEs and all are known to be associated with PegIFNα-2a/RBV treatment. The majority of AEs were Grade 1 or 2 in severity. There were also no unexpected observations in terms of the frequency or severity of the reported events of special/clinical interest. Decrease in neutrophil and precursor count was the only graded treatment-emergent laboratory abnormality for which Grade 3 or 4 abnormalities were observed in >10% of patients (19.3%).

In the Phase 1 group, half of the patients (8/16) had not been previously exposed to simeprevir in a Phase 1 study and, of these, the majority (6/8, 75%) were prior non-responders to PegIFN/RBV treatment. The SVR12 rate was 37.5% (6/16); all patients who achieved SVR12 also went on to achieve SVR24.

Equal proportions of patients with and without prior exposure to simeprevir achieved SVR12 (3/8 patients each). Among the 8 subjects who were not previously exposed to simeprevir, 2 of the 6 prior non-responders to PegIFN/RBV treatment achieved SVR12, one prior relapser achieved SVR12, and one treatment-naïve patient did not achieve SVR12. Patients with prior simeprevir exposure were treatment-naïve or prior relapsers to PegIFN/RBV. Given the small sample size in these subgroups, it is difficult to draw conclusions for a comparison of the SVR rates to historic controls.

Multiple factors may have contributed to the lower virologic response rate in the Phase 1 group. Missing data at the SVR12 assessment time point was the reason for post-treatment failure in 12.5% of subjects (2/16) in the Phase 1 group. In addition, among the group of 8 patients in the Phase 1 group who had not previously been exposed to simeprevir, 1 patient (prior non-responder) discontinued treatment early at Week 36 due to AEs and 2 patients discontinued treatment early due to consent withdrawal (one prior non-responder on Day 5 and one simeprevir-naïve patient at Week 16). None of these 8 simeprevir-naïve patients had the favorable baseline *IL28B* CC genotype. Among the group of 8 patients in the Phase 1 group who had previously been exposed to simeprevir, 2 subjects discontinued treatment early due to AEs. One of these 8 simeprevir-experienced patients had the favorable *IL28B* CC genotype and this patient achieved SVR12. Of note, the R155K substitution was found emerging in 6/8 patients during previous short-term exposure to simeprevir in the Phase 1 study and was still detected in 2 patients using population sequencing at baseline in the current study but may have still been present as minority substitution in other patients at baseline. The presence of this substitution could have also impacted the treatment response in this group of simeprevir-experienced patients.

The proportion of patients with on-treatment failure (18.8%) was similar to that of the Phase 2/3 group. The incidence of viral breakthrough was <10% and 10/12 patients with viral breakthrough were prior null responders to PegIFN/RBV treatment.

A limitation of the study was the small sample size in the Phase 1 group (*n* = 16). In addition, when the Phase 2/3 group was analyzed according to response to prior treatment, patient numbers were relatively low in some of the groups (viral breakthrough, *n* = 10, partial responder, *n* = 28, null responder, *n* = 30). Similar limitations in terms of patient numbers also applied to other subgroups, for example, grouping according to *IL28B* genotype.

## Conclusions

Treatment with simeprevir 150 mg QD in combination with PegIFNα-2a/RBV for 12 weeks allowed the majority of RGT-eligible patients to achieve SVR12 while the shortening of treatment to 24 weeks in total. As previously described, simeprevir with PegIFNα-2a/RBV achieved a lower SVR rate among patients with HCV GT1 infection who were prior non-responders, compared to different patient populations that have previously been described in other studies [12–14]. The safety profile of simeprevir with PegIFNα-2a/RBV in this small study was consistent both with that described in the approved labels for simeprevir and with that reported in previous large Phase 3 studies.

These results provide yet further evidence to support the added potency that simeprevir offers as part of an IFN-containing regimen. However, the role of simeprevir plus PegIFNα/RBV as the primary treatment for patients with HCV GT1 infection has diminished since the recent development of IFN-free DAA combinations, which provide a shorter, more effective, and better tolerated treatment across most patient populations.

Retreatment of virologic failures to simeprevir plus PegIFNα/RBV will require regimens which include 2 or more DAAs without cross-resistance such as an NS5A inhibitor and NS5B inhibitor. Highly effective retreatment options are currently available for patients who have failed prior IFN-free or IFN-containing DAA HCV therapy [18, 20].

## Additional files

Additional file 1: Table S1. Baseline characteristics and early response parameters with a statistically significant effect on SVR12 in multivariate analyses in the Phase 2/3 group (ITT population). (DOCX 22 kb)

Additional file 2: List of ethics committees. (DOCX 67 kb)

## Abbreviations

AE: Adverse event
ALT: Alanine aminotransferase
AST: Aspartate aminotransferase
BMI: Body mass index
cEVR: Complete early virologic response
CI: Confidence interval
DAA: Direct-acting antiviral agent
EOT: End of treatment
EOTR: End of treatment response
EU: European Union
GT: Genotype
HCV: Hepatitis C virus
HIV: Human immunodeficiency virus
HOMA-IR: Homeostatic model assessment of insulin resistance
*IL28B*: Interleukin-28b
ITT: Intent-to-treat
NS5B: Non-structural protein 5B
PegIFNα-2a: Peginterferon α-2a
QD: Once daily
RBV: Ribavirin
RGT: response-guided therapy
RVR: Rapid virologic response
SAE: Serious adverse event
SD: Standard deviation
SE: Standard error
SMV: Simeprevir
SVR12: Sustained virologic response 12 weeks after planned end of treatment
SVR24: Sustained virologic response 24 weeks after planned end of treatment
SVR4: Sustained virologic response 4 weeks after planned end of treatment
WHO: World Health Organization
