# Supplementary material for: Simeprevir with peginterferon α-2a/ribavirin for chronic hepatitis C virus genotype 1 infection in treatment-experienced patients: an open-label, rollover study
Source: BMC Infect Dis. 2017 Jun 2;17:389. doi: 10.1186/s12879-017-2444-3 (PMC5457573; doi:10.1186/s12879-017-2444-3)
Supplement: Supplementary file 1 — Baseline characteristics and early response parameters with a statistically significant effect on SVR12 in multivariate analyses in the Phase 2/3 group (ITT population). (DOCX 22 kb) [file 12879_2017_2444_MOESM1_ESM.docx]

**Additional file 1**

**Table S1.** Baseline characteristics and early response parameters with a statistically significant effect on SVR12 in multivariate analyses in the Phase 2/3 group (ITT population)

|  | P-value^a^ |
| --- | --- |
| Baseline HOMA-IR | 0.022 |
| Prior PegIFN/RBV Response | 0.009 |
| cEVR | <0.001 |
| HCV RNA <25 IU/ml at Week 4 | 0.009 |

^a^ Likelihood ratio test

cEVR, complete early virologic response, *HCV* hepatitis C virus, *HOMA-IR* homeostatic model assessment of insulin resistance, *ITT* intent-to-treat, *PegIFN/RBV* peginterferon with ribavirin, *RNA,* ribonucleic acid, *SVR12* sustained virologic response 12 weeks after planned end of treatment
