# Supplementary material for: Simeprevir with peginterferon α-2a/ribavirin for chronic hepatitis C virus genotype 1 infection in treatment-experienced patients: an open-label, rollover study
Source: BMC Infect Dis. 2017 Jun 2;17:389. doi: 10.1186/s12879-017-2444-3 (PMC5457573; doi:10.1186/s12879-017-2444-3)
Supplement: Supplementary file 2 — List of ethics committees. (DOCX 67 kb) [file 12879_2017_2444_MOESM2_ESM.docx]

**Ethics approval and consent to participate**

| **Country** | **IRB/EC Name/Address** |
| --- | --- |
| Argentina | 1. Comité de Ética en Farmacología Clínica de la Fundación CIDEA – CEFC Paraguay 2041 piso 9 departamento “H” (C1121ABE) Ciudad Autónoma de Buenos Aires – Argentina 2. Comité Independiente de Ética para Ensayos en Farmacología   Clínica J. E. Uriburu 774, 1° piso (C1027AAP) Ciudad Autónoma de Buenos Aires – Argentina   1. Comité de Ética del Hospital Provincial del Centenario Urquiza 3101 (S2002KDS) Rosario – Argentina |
| Australia | 1. Royal Adelaide Hospital Research Ethics Committee Royal Adelaide Hospital North Terrace Infectious Diseases Laboratory Adelaide, SA, 5000 Australia 2. Alfred Human Research & Ethics Committee Alfred Hospital Commercial Rd Melbourne, VIC, 3004 Australia 3. Sydney West Area Health Service HREC Westmead Hospital Darcy Road Westmead Sydney, NSW, 2145 Australia 4. Metro South Health Service District Human Research Ethics Committee Princess Alexandra Hospital 199 Ipswich Road Woolloongabba, Qld, 4102 Australia |
| Austria | 1. Ethikkommission d. Med. Universität Wien und des Allgemeinen Krankenhauses der Stadt Wien Borschkegasse 8B/E 06 Wien, 1090 Austria |
| Belgium | 1. UCL Saint-Luc 10 Avenue Hippocrate Brussel, B-1200 Belgium 2. Commissie Medische Ethiek UZ Leuven Herestraat 49 UZ Leuven Leuven, 3000 Belgium 3. Commissie Medische Ethiek AZ-VUB Laarbeeklaan 101 JETTE, 1090 Belgium 4. Commissie Medische Ethiek UZ Gent De Pintelaan 185 Gent, 9000 Belgium 5. Ethische Commissie AZ Sint-Jan Ruddershove 10 Brugge, 8000 Belgium 6. Comité d'ethique Hôpital Erasme Hôpital Erasme Route de Lennik 808 Brussel, 1070 Belgium 7. Commissie voor Medische Ethiek - ZNA/OCMW Antwerpen Lindendreef 1 ZNA Koningin Paola Kinderziekenhuis P6 lokaal 617 Antwerpen, 2020 Belgium |
| Brazil | 1. Comite De Etica Em Pesquisa Complex Hospitalar Universitario Professor Edgard Santos Rua Augusto Viana s/n Canela Salvador, 40110-160 Brazil 2. Comite De Etica Em Pesquisa Em Seres Humanos Do Hospital Heliopolis Rua Conego Xavier, 276 10 Andar Sacoma Sao Paulo, 55 11 2215-1001, 04231-030 Brazil 3. CONEP - Comissao Nacional de Etica em Pesquisa SEPN 510 NORTE BLOCO A 1º SUBSOLO – EDIFÍCIO EXINAN Brasilia Brazil 4. Comite de Etica em Pesquisa em Seres Humanos da UNIFESP Rua Botucatu, 572 – 1º Andar / CJ. 14 Sao Paulo Brazil 5. CAPPesq - Comissao de Etica Analise Projetos Pesquisa Rua Ouvideo Pires de Campos, 255 sl 505 Sao Paulo, SP Brazil 6. Comite De Etica Em Pesquisa-CRT/DST-AIDS Rua Santa Cruz 81 AMBULATÓRIO SALA 11A Sao Paulo, SP, 04121-000 Brazil |
| Bulgaria | 1. Ethics Committee For Multicenter Trials 5 Sveta NedelyaSq. Ministry of Health Sofia, 1000 Bulgaria |
| Canada | 1. Ottawa Hospital Research Ethics Board 751 Parkdale Ave Suite 106 CIVIC CAMPUS Ottawa, ON, K1Y 1J7 Canada 2. Providence Health Care Research Ethics Board 1081 Burrard Stcomox Building Room B180-35 Vancouver, BC, V6Z 1Y6 Canada 3. University Health Network Research Ethics Board 700 University Avenue 8^th^ Floor South-Room 8-18 Toronto, ON, M5G 1Z5 Canada 4. McGill University Health Centre Research Ethics Board 1650, av. Cedar Montreal, PQ, H3G 1A4 Canada 5. Veritas IRB 1255 Transcanada Hwy Suite 310 Dorval Montreal, QC, H9P 2V4 Canada 6. Conjoint Health Research Ethics Board Dr. Glenys Godlovitch IRB/IEC, Chairman 3330 Hospital Dr Nw Room 3330 Calgary, AB, T2N 4N1 Canada |
| France | 1. CPP SUD-EST III Groupement Hospitalier Est Batiment Pinel 59 Boulevard Pinel 69500 Bron France 2. CPP SUD-EST III Groupement Hospitalier Edouard Herriot Batiment 12 - 1er étage 5, Place d'Arsonval Lyon CEDX 03, 69437 France |
| Germany | 1. Landesamt für Gesundheit und Soziales Berlin Geschaeftsstelle der Ethik-Kommission des Landes Berlin Fehrbellinerplatz 1 LaGeSo Berlin Geschäftsstelle der EK des Landes Berlin Berlin, 10707 Germany 2. Ethik-Kommission Der Medizinischen Fakultät Der Universität Würzburg Institut für Pharmakologie und Toxikologie Versbacher Strasse 9 Würzburg, 97078 Germany 3. Ethik-Kommission der Ärztekammer Schleswig-Holstein Bismarckallee 8-12 Bad Segeberg, 23795 Germany 4. Ethik-Kommission Der Ärztekammer Nordrhein Tersteegenstr 9 Düsseldorf, 40474 Germany 5. Ethik-Kommission des Fachbereichs Medizin der Johann-Wolfgang-Goethe Universität Theodor-Stern-Kai 7 HAUS 1 Frankfurt, 60590 Germany 6. Ethik-Kommission Der Ärztekammer Hamburg Weidestr. 122b Hamburg, 22083 Germany 7. Ethik-Kommission Der Universität Ulm Helmholtzstraße 20 (Oberer Eselsberg) Ulm, 89081 Germany 8. Ethikkommission Der Ärztekammer Westfalen-Lippe Und Der Med. Fakultät der wwu-münster Gartenstr. 210-214 Muenster, 48147 Germany |
| Great Britain | 1. London - South East. South East Coast Strategic Health Authority Preston Hall Aylesford, Kent, ME20 7NJ Great Britain |
| Israel | 1. Helsinki Committee – Rambam Medical Center Ha'aliyah Street Bat Gallm Haifa, 31096 Israel 2. Helsinki Committee – Sourasky Medical Center 6 Weitzman Street Tel Aviv, 64239 Israel 3. Helsinki Committee – Lady Davis Carmel Medical Center 7 Michal Street Gastroenterology unit HAIFA, 34362 Israel 4. Helsinki Committee – Rebecca Ziv Medical Center Darom Neighborhood Zefat, 13000 Israel 5. Helsinki Committee – Chaim Sheba Medical Center Tel Hashomer Ramat Gan, 52621 Israel 6. Helsinki Committee – Holy Family Medical Center Holly Family Medical Center Nazareth Israel |
| Italy | 1. Comitato Di Etica - Fondazione Irccs Ca' Granda Ospedale Maggiore Policlinico Via Francesco Sforza 28 Milano, 20122 Italy 2. Comitato Etico Dell'azienda Ospedaliera Spedali Civili Di Brescia Piazzale Spedali Civili 1 Brescia, 25123 Italy 3. Comitato Etico Dell`Azienda Ospedaliera Universitaria Policlinico Paolo Giaccone Via Del Vespro 127 Palermo, PA, 90127 Italy 4. Comitato Etico Dell`azienda Ospedaliera Universitaria S. Giovanni Battista Di Torino Corso Bramante 88/90 Torino, TO, 10126 Italy 5. Comitato Etico Indipendente Dell´azienda Ospedaliero Universitaria Policlinico S. Orsolamalpighi Di Via Albertoni 15 Bologna, 40138 Italy 6. Comitato Etico Dell'azienda Policlinico Umberto I Di Roma Viale Del Policlinico 155 Roma, 00161 Italy 7. Comitato Di Bioetica Della ASL Di Sassari Via Monte Grappa N. 82 07100 Sassari (Sassari) |
| Mexico | 1. Comite Institucional De Investigacion Biomedica En Humanos Vasco de Quiroga No. 15, Del. Tlalpan Distrito Federal, 14000 Mexico 2. Comite De Etica Secretaria De Investigacion Facultad De Medicina Uanl Aguirre Pequeño S/N Mitras Centro Monterrey, 64460 Mexico 3. Comite De Etica Del Instituto Jalisciense De Investigacion Clinica Penitenciaria No.20, Col. Centro Guadalajara, 44100 Mexico |
| Netherlands | 1. Academisch Medisch Centrum, METC Postbus 22660 Amsterdam, 1100 DD Netherlands 2. METC ERASMUS MC Postbus 2040 Rotterdam, 3000 CA Netherlands 3. EC Leiden University Medical Center Albinusdreef 2 Leiden, 2333ZA Netherlands |
| New Zealand | 1. Northern Y Ethics Committee 3rd Floor Unisys Building 650 Great South Road Auckland New Zealand |
| Poland | 1. Komisja Bioetyczna Przy Slaskiej Izbie Lekarskiej Ul Grazynskiego 49A Katowice, 40-126 Poland |
| Portugal | 1. Comissão de Ética para a Investigação Científica Parque De Saude De Lisboa Avenida Do Brasil 53 PAV. 17 – A Lisboa, 1749004 Portugal |
| Puerto Rico | 1. Western Institutional Review Board 3535 Seventh Avenue SW Olympia, WA 98502-5010 USA 2. Sterling Institutional Review Board 6300 Powers Ferry Road Suite 300-351 Atlanta, GA 30339 USA |
| Romania | 1. National Bioethics Committee of the Medicines and Medical Devices 21 Stefan cel Mare Av, 020125 Bucharest, Sector 2, Romania |
| Russia | 1. Ind. Interdisc. Comm. On Ethical Exp. of CLN Studies 51 Leningradsky Prospekt Moscow, 125468 Russia 2. Independent Ethics Committee Of Smolensk Regional Clinical Hospital 27 Gagarina Avenue Smolensk, 214019 Russia 3. Independent Ethics Committee Of Moscow Regional Clinical Inst Vladimirsky 61/2 Schepkina Street Moscow, 129111 Russia 4. Interuniversity Ethics Committee And Assoc Of Medical And Pharmaceutical Inst Of Higher Education 8 Build 2 Trubetskaya Str Moscow, 119992 Russia 5. Interuniversity Ethics Committee And Assoc Of Medical And Pharmaceutical Inst Of Higher Education 8 Build 2 Trubetskaya Str Moscow, 119992 Russia 6. Ethics Committee Of City Clinical Hospital #64 61 Vavilova Str Moscow, 117292 Russia 7. Ethical Committee Of Stavropol State Medical Academy Roszdrava 310, Mira Street Stavropol, 355017 Russia |
| Spain | 1. Comite Etico De Investigacion Clinica Del Hospital Universitari Vall D'hebron - 2a Planta Paseo Vall D'hebron, 119-129 Institut De Recerca Hospital Universitari Vall D'hebron Barcelona, 08035 Spain 2. CEIC Area 6 – Hospital Universitario Puerta De Hierro De Majadahonda C/ Joaquin Rodrigo, 2 Nefrologia Majadahonda (Madrid), 28222 Spain 3. CEIC Hospital La Ribera de Alzira Carretera Corbera, Km. 1 Hospital La Ribera Alzira, Valencia, 46600 Spain 4. CEIC Autonómico De Ensayos Clínicos De Andalucía Consejería De Salud Avda. Innovación s/n Eidf. Arena 1 Sevilla, 41020 Spain 5. EC Of Hospital De Valme Carretera De Cadiz S/n Km 548 9 CEIC Hospital de Valme Sevilla, 41014 Spain 6. Hospital Clinic I Provincial Comite Etico De Investigacion Clinica C/ Villarroel, 170 Agencia De Ensayos Clínicos Barcelona, 08036 Spain |
| Turkey | 1. Ege Unversity Medical Faculty Ethics Committee Bornova-Izmir Izmir, Izmir, 35100 Turkey |
| Ukraine | 1. Central Ethics Commission of MoH of Ukraine 5 Narodnogo Opolchennya Str Msp Kiev, 03680 Ukraine 2. Ethics Commission of City Clinical Hospital № 1 96 Khmelnitske shosse Vinnitsa, 21029 Ukraine 3. Ethic Committee of Central City Clinical Hospital #1 of Donetsk City 52-A, Rosa Luksemburh Str. Donetsk, 83114 Ukraine 4. Ethic Committee of Kyiv City Clinical Hospital #9 1, Ryzka Str. Kyiv, 04112 Ukraine 5. Ethics Committee of the Kyiv City Clinical Hospital № 5 11 Vidpochynku Str Kyiv, 03115 Ukraine |
| USA | 1. Sterling Institutional Review Board 6300 Powers Ferry Rd. Atlanta, GA, 30339 USA 2. Ochsner Clinical Foundation Institutional Review Board 1514 Jefferson HWY New Orleans, LA, 70121 USA 3. Baylor Research Institute 3310 Live Oak Suite 501 Dallas TX 75246 USA 4. Western Institutional Review Board 3535 Seventh Ave SW Olympia WA 98502 USA 5. Cedars Sinai Medical Center IRB 8383 Wilshire Blvd SUITE 305 Beverly Hills, CA, 90211 USA 6. Weill Cornell Medical College IRB 407 East 61st Street 1st Floor New York, NY, 10065 USA 7. University of North Carolina at Chapel Hill - Office of Human Research Ethics Medical School Building 52 - Mason Farm Road - CB # 7097 Chapel Hill, NC, 27599 USA 8. University of Pennsylvania 3624 Market Street - ST. 301-S Suite 301 S Philadelphia, PA, 19104 USA 9. The University of Chicago IRB 5751 S Woodlawn Ave McGiffert Hall, 2nd floor Chicago, IL, 60637 USA 10. North Mississippi Health Services IRB 830 South Gloster Street East Tower 3rd Floor Tupelo, MS, 38801 USA 11. Cooper Green Mercy Hospital IRB 1515 6TH Ave S. Birmingham, AL, 35233 USA |
